# First Isolation of the Heteropathotype Shiga Toxin-Producing and Extra-Intestinal Pathogenic (STEC-ExPEC) *E. coli* O80:H2 in French Healthy Cattle: Genomic Characterization and Phylogenetic Position

**DOI:** 10.3390/ijms25105428

**Published:** 2024-05-16

**Authors:** Nathan Soleau, Sarah Ganet, Stéphanie Werlen, Lia Collignon, Aurélie Cointe, Stéphane Bonacorsi, Delphine Sergentet

**Affiliations:** 1 ‘Bacterial Opportunistic Pathogens and Environment’ (BPOE) Research Team, UMR5557 Ecologie Microbienne Lyon, CNRS (National Center of Scientific Research), VetAgro Sup, Université de Lyon, Marcy-l’Étoile, 69280 Lyon, France; nathan.soleau@vetagro-sup.fr (N.S.); sarah.ganet@vetagro-sup.fr (S.G.); 2 Laboratoire d’Étude des Microorganismes Alimentaires Pathogènes–French National Reference Laboratory for *Escherichia coli* Including STEC (NRL-STEC), VetAgro Sup–Campus Vétérinaire, Université de Lyon, Marcy-l’Étoile, 69280 Lyon, France; 3 Service de Microbiologie, Centre National de Référence *Escherichia coli*, AP-HP, Hôpital Robert-Debré, Université Paris-Cité, IAME, UMR 1137, INSERM, 75018 Paris, France; aurelie.cointe@aphp.fr (A.C.); stephane.bonacorsi@aphp.fr (S.B.)

**Keywords:** Shiga-toxin-producing *Escherichia coli* (STEC), first isolation, characterization, whole-genome sequencing, serotype O80:H2, emerging pathogen

## Abstract

The emerging heteropathotype shigatoxigenic (STEC) and extra-intestinal pathogenic *Escherichia coli* (ExPEC) O80:H2 has been the second leading cause of pediatric HUS in France since the mid-2010s. In contrast with other highly pathogenic STEC serotypes, for which ruminants have clearly been identified as the main human infection source, this heteropathotype’s reservoir remains unknown. In this context, we describe for the first time the isolation of seven STEC O80:H2 strains from healthy cattle on a single cattle farm in France. This study aimed at (i) characterizing the genome and (ii) investigating the phylogenetic positions of these O80:H2 STEC strains. The virulomes, resistomes, and phylogenetic positions of the seven bovine isolates were investigated using in silico typing tools, antimicrobial susceptibility testing and cgMLST analysis after short-read whole genome sequencing (WGS). One representative isolate (A13P112V1) was also subjected to long-read sequencing. The seven isolates possessed ExPEC-related virulence genes on a pR444_A-like mosaic plasmid, previously described in strain RDEx444 and known to confer multi-drug resistance. All isolates were clonally related and clustered with human clinical strains from France and Switzerland with a range of locus differences of only one to five. In conclusion, our findings suggest that healthy cattle in France could potentially act as a reservoir of the STEC-ExPEC O80:H2 pathotype.

## 1. Introduction

Shigatoxigenic *Escherichia coli* (STEC) constitutes a group of foodborne pathogens capable of causing human diseases ranging from acute diarrhea to severe complications, including hemolytic and uremic syndrome (HUS). Shiga toxins (Stx) are the major virulence factor in STEC, and an association has been observed between the type or subtype of Stx and the severity of human disease. Several studies have shown that certain subtypes of this toxin, such as Stx2a and 2d, are frequently associated with a higher risk of inducing HUS than Stx1 and other Stx2 subtypes [1]. Highly pathogenic STEC strains also generally harbor the *eae* gene, coding for an adhesin called intimin [2,3]. This virulence factor is involved in the adhesion to the gastro-intestinal tract and is responsible for attaching and effacing lesions in enterocytes [4].

Out of the hundreds of documented STEC serotypes, only a minority are associated with the majority of human infections. In France, historically, the STEC serotypes most associated with human infection cases have been O26:H11, O103:H2, O111:H8, O145:H28, and O157:H7, also called the “Top 5 serotypes” [5]. In the early 2010s, the new heteropathotype Shiga-toxin-producing and extra-intestinal pathogenic (STEC-ExPEC) *E. coli* O80:H2 emerged in France, and it has since been responsible for an increasing number of pediatric HUS cases, some of which are associated with bacteremia and other forms of severe infection [6,7]. Since 2015, STEC O80:H2 has been the second leading cause of HUS in France after O26:H11 and ahead of the historically most prevalent serotype O157:H7 [8,9]. In 2021, even at the European level, the serogroup O80:H2 was ranked third among the most common STEC serogroups implicated in HUS cases [10]. Previous genomic characterizations of O80:H2 STEC strains revealed the presence of the uncommon *eae*-ξ intimin subtype and the presence of a mosaic plasmid combining virulence and antimicrobial resistance (AMR) genes [6,11,12]. Indeed, the plasmid pR444_A, previously described in the human O80:H2 STEC strain RDEx444, is a pS88-like plasmid combining extra-intestinal virulence genes characteristic of ExPEC strains (known to be involved in neonatal meningitis) and an additional antimicrobial resistance cassette [12]. Phylogenetic investigations conducted on O80:H2 STEC and entero-pathogenic (EPEC) strains isolated in France, Belgium, the United Kingdom and Italy showed that all O80:H2 strains were closely related regardless of their pathotype and that they all belonged to the sequence type ST301 [12,13,14,15,16].

In Europe, STEC strains are mainly transmitted to humans through contaminated food, with animal products being the most at risk [10]. Cattle, as asymptomatic carriers, have been reported to be the primary source of food contamination, either directly or indirectly through the environment [17]. However, the reservoirs and sources of human contamination by STEC O80 strains remain unclear, hence the lack of routine surveillance of this serotype in foodstuffs by food business operators. In France, three O80:H2 STEC strains (511-4, H39-78, and H15-66.3) were isolated from raw cow milk cheese by the French National Reference Laboratory (FNRL) in 2013 and 2014 [7,12], suggesting that foodstuffs are potential sources of infection and, upstream, that there is a potential for shedding by cattle. However, from 2016 to 2022, the French national monitoring program for STEC in foodstuffs carried out by the French ministry of agriculture and the NRL failed to detect STEC O80:H2 from among 3900 examined food samples [18]. Likewise, according to the “European Union One Health 2021 Zoonoses report”, serogroup O80 was not found among all STEC strains isolated from foodstuffs in 2021 [10]. 

Considering the severe nature of O80:H2 infections in humans, it appears to be of the utmost importance to continue gathering data on potential reservoirs of this pathotype in order to implement adapted control measures and surveillance. Recent studies have attempted to bring forward evidence of STEC O80:H2 cattle carriers, yielding limited results aside from nine STEC O80:H2 isolates recovered from diarrheic and septicemic calves in a Belgian study [15]. To the best of the authors’ knowledge, STEC O80:H2 has never been isolated from healthy or symptomatic cattle in France [10,11,12,14], and the source of human infection by this emerging heteropathotype remains to be fully clarified. 

Our study describes, for the first time, the isolation of seven STEC O80:H2 strains from seven distinct individuals (five cows and two calves) during an ongoing longitudinal survey primarily investigating the “top 5” STEC carriers among cattle on a single French cattle farm. This study aimed at (i) characterizing the genomes and (ii) investigating the phylogenetic positions of seven O80:H2 STEC strains isolated from healthy cattle. 

## 2. Results

### 2.1. Bacterial Strains

STEC O80:H2 isolates A10AV1, A13P112V1, A15P129V1, A23P11V1, and A29P14V1, isolated from five healthy cows, and isolates VX1P21V1 and VX4P17V1, obtained from two healthy calves, were obtained on a single French cattle farm in May 2023. These seven isolates were recovered from 7 out of 44 animals (15.9%) present at the time of sampling. 

Also, five additional human clinical strains isolated between 2022 and 2023 were provided by the French National Reference Center for *E. coli* (University Hospital Robert-Debré) for sequencing and subsequent genomic comparison with the above-mentioned bovine isolates. 

### 2.2. Clonal Relationships of French Bovine STEC O80:H2

All seven bovine isolates considered in this study belonged to ST301 and CC165 and were highly related based on core genome multi-locus sequence typing (cgMLST) analysis. Only the A10AV1 isolate differed from the others by one cgMLST allele. Isolate A13P112V1 was considered a representative of all strains and was selected to further characterize the genomic content and structures of the isolates.

### 2.3. Genomic Structure and Genetic Features of French Bovine Isolates

The hybrid assembly of A13P112V1 sequencing reads yielded five circularized contigs including the chromosome and four plasmids with lengths ranging from 52,166 bp to 156,714 bp (e.g., “base pairs”). 

All strains carried the *stx2d* variant of the Shiga-toxin-encoding gene and a complete locus of enterocyte effacement (LEE) harboring the rare *eae-ξ* variant of the intimin-encoding gene. According to the in silico virulotyping results, all the isolates were positive for 11 genes associated with extra-intestinal virulence and carried by the pR444_A plasmid of the previously described strain RDEx444 [12]: *cia*, *cvaC*, *etsC*, *hlyF*, *iroN*, *iss*, *iucC*, *iutA*, *mchF*, *ompT_p_*, and *sitA.* The EHEC-associated enterohemolysin (*ehxA)*, serine protease (*espP*), and colicins A (*cia*) and M (*cma*) were also found in the genomes of all strains.

In terms of antibiotic resistance genes, the strains analyzed herein possessed multiple antimicrobial resistance (AMR)-encoding regions. Specifically, all seven isolates encoded at least six plasmid-located antibiotic resistance genes conferring resistance to streptomycin (*aph(3″)-Ib, aph(6)-Id*), kanamycin (*aph(3′)-Ia*), beta-lactam (*bla_TEM-1_*), and sulfamethoxazole/trimethoprim (*sul*, *dfrA*). The tetracycline-resistance-associated gene *tetA* was present in all but one isolate (A10AV1), and the mercury-resistance-conferring *mer* operon was identified in every isolate. Also, they all carried the chromosomal p.S83L mutation in the DNA gyrase *gyrA* gene known to be responsible for quinolone resistance [19].

**Figure 1:**
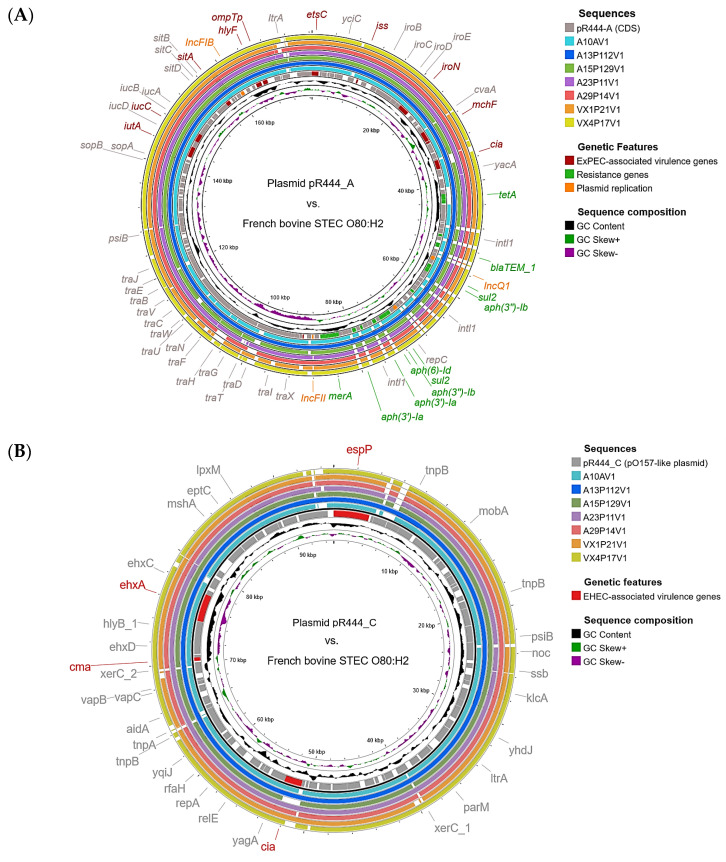
Comparison of reference plasmids from STEC-ExPEC O80:H2 strain RDEx444 with the whole genomes of the seven French bovine isolates using BLASTN on the Proksee platform. (**A**) The innermost ring corresponds to the reference sequence of pR444_A, with only the coding sequences showcased. Annotation was performed using the Prokka tool (v.1.14.6) [21] and the pR444_A-corresponding annotation multi-fasta file retrieved from GenBank (accession number: NZ_QBDM01000004.1). (**B**) The sequence of the pO157-like plasmid pr444_C was used as a reference for alignment (accession number: NZ_QBDM01000002.1), and annotation was performed as described above. The genome sequence of isolate A13P112V1 was generated via hybrid assembly after both long-read and short-read sequencing. The other genomic sequences of the other isolates were generated via SPAdes assembly [22] after short-read sequencing only. Genetic features of interest are highlighted according to the colored legend on the right. Missing genomic regions compared to the reference plasmids are represented by white gaps within the colored rings.

Whole-genome comparisons with the reference plasmid pR444_A conducted using the Proksee online platform [20] (Figure 1A) further confirmed the presence of a pR444_A-like mosaic plasmid carrying both extra-intestinal virulence genes and a multi-drug-resistance-encoding region in these isolates. They also showed the presence of a pO157-like plasmid harboring enterohemorrhagic-associated (EHEC) virulence genes (Figure 1B) and a non-conjugative cryptic plasmid, all of which have been previously described in strain RDEx444. The fourth plasmid of isolate A13P112V1 possessed an incomplete set of genes involved in plasmid transfer but did not possess any virulence-associated or AMR-associated genes.

### 2.4. Antimicrobial Susceptibility Profiles of French Bovine STEC O80:H2

The testing of all seven bovine strains for susceptibility to antibiotics with both veterinary and clinical relevance revealed a multidrug resistance profile matching the previous in silico resistome characterization. Indeed, these isolates were resistant to at least one antimicrobial drug in three or more antimicrobial classes. Specifically, all but one isolate were resistant (Table 1) to the following antimicrobials: amoxicillin (AMX), streptomycin (S), kanamycin (K), tetracycline (TET), sulfamethoxazole/trimethoprim (SXT), flumequine (UB), and nalidixic acid (NAL). Isolate A10AV1, which lacked the tetracycline resistance gene *tetA*, was found to be susceptible to tetracycline. 

### 2.5. Phylogenetic Positions of French Bovine STEC-ExPEC O80:H2 Isolates among European STEC-ExPEC O80:H2 Strains

**Figure 2:**
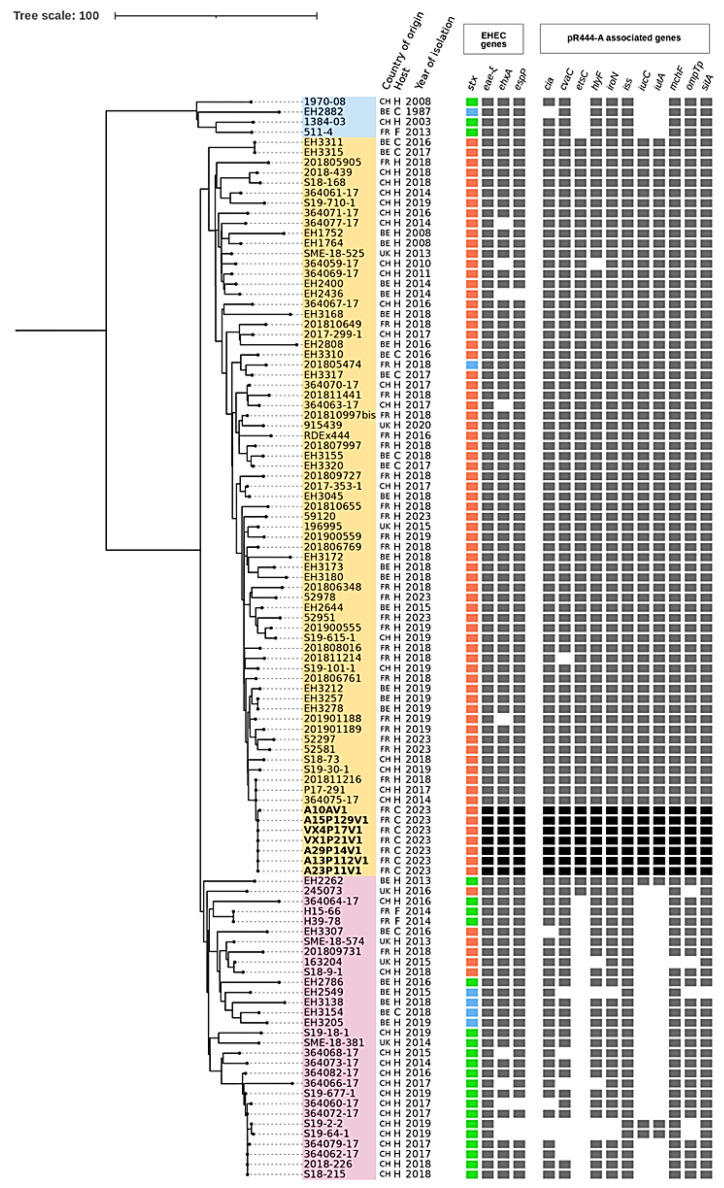
Phylogenetic tree based on cgMLST analysis of 107 STEC-ExPEC O80:H2 specimens isolated from various sources and geographic origins in Europe between 1997 and 2023 [12,15,25,26]. Darkly highlighted isolates are the seven bovine isolates from this study. Clade 1 is highlighted in light blue, and subclades 2.1 and 2.2 are highlighted in yellow and pink, respectively. The geographic origins of the strains are indicated by suitable abbreviations (BE, Belgium; CH, Switzerland; FR, France; UK, United Kingdom). Sources of origin are represented by the following codes: H, human; C, cattle; F, foodstuffs (raw milk cheese). The subtypes of the Shiga toxin gene (*stx*) are represented by different colored squares (stx1a, blue; stx2a, green; stx2d, orange). The presence of virulence-associated genes is indicated by grey or black squares.

In order to investigate the relatedness of the seven bovine isolates with respect to other STEC O80:H2 strains, we conducted a core-genome-based phylogenetic analysis using a set of 100 O80:H2 STEC strains previously published or available on public databases (Enterobase and Genbank) from different hosts and geographic origins. The number of locus differences in the collection ranged from 0 to 165. All strains were classified as ST301, and core-genome Multi Locus Sequence Typing (cgMLST) analysis distinguished two main clades (Figure 2), with clade 1 including only four strains isolated from 1987 to 2013 and clade 2 including strains isolated between 2008 and 2023. 

Clade 2 was divided into two subclades. Subclade SC2.1 comprised strains carrying almost exclusively *stx2d* and a complete set of pR444_A-associated virulence genes. The strains originated from several geographic areas, and their isolation period ranged from 2008 to 2023. Most of the French isolates, including the seven bovine isolates, were grouped into the latter subclade. In contrast, subclade SC2.2 comprised mostly *stx2a*-carrying strains and an incomplete pR444_A-like plasmid (i.e., lacking at least one of the following genes: *etsC*, *iucC*, and/or *iutA*). Most strains in the clade originated from Belgium and Switzerland, while only 3 out of the 30 strains were isolated in France. Based on the clustering of the French bovine clones with clinical isolates (364075-17 and P17-291 from Switzerland and 201811216 from France), this phylogenetic tree illustrates the high proximity between the bovine and human strains.

## 3. Discussion

First described in France in 2005 following a raw-milk-cheese-consumption-related HUS outbreak [27,28], the STEC-ExPEC O80:H2 heteropathotype has been increasing in prevalence in France since the 2010s and recently spread to other European countries [6,8,15,16,25,29,30,31,32]. Since 2015, this serotype has even been the second leading cause of pediatric HUS in France, with several severe cases reported in the literature both in France and the rest of Europe [6,8,29].

So far, only a dozen isolates have been recovered from animals, almost exclusively from septicemic and diarrheic calves [11,14,15], and from foodstuffs (raw milk products) [7,31]. The few attempts to isolate STEC O80:H2 from healthy cattle were unfruitful [15,33]. An investigation of factors associated with STEC O80:H2 infections in France conducted by Santé Publique France demonstrated that patients infected with this pathotype were less likely to have consumed ground beef compared with those infected with O157:H7 and other serotypes. This study suggested a lesser role of the cattle reservoir in human infections with STEC O80:H2 and the existence of different reservoirs and sources of infection [30].

In this study, we described, for the first time, the isolation of STEC-ExPEC O80:H2 in healthy adult cattle and calves from a single cattle farm in France. On the studied farm, a total of 7/44 (15.9%) animals present at the time of sampling shed STEC O80:H2 in their feces, and all seven isolates were considered to be clonally related based on cgMLST analysis, suggesting interindividual transmission within the broad farm environment or a common origin of this contamination, such as contaminated feed or water.

Similar to other described STEC strains and EPEC O80:H2 [13,15,31,32] and in the absence of any known epidemiological link, a broader comparative phylogenetic analysis revealed high genetic proximity between STEC O80:H2 described in our study and STEC O80:H2 isolated from humans. Indeed, the seven French bovine STEC strains belong to a subclade comprising *stx2d*-carrying strains isolated from humans and cattle in multiple European countries. More specifically, the seven studied isolates clustered (i.e., displayed fewer than five cgMLST locus differences) with two human STEC O80:H2 isolates from Switzerland isolated in 2014 and 2017 and with one human clinical isolate from France dating back to 2018.

Interestingly, our bovine isolates were recovered from a cattle farm located in one of the high-incidence regions of France described by Santé Publique France regarding STEC-O80:H2-associated infections [7,30]. On the basis of the peculiar geographic distribution of STEC O80:H2 human infections and the admittedly limitedly coinciding location of the newfound bovine isolates, these data raise questions about the existence of a region-wide reservoir of this pathotype in France.

Based on in silico investigations of the genomic content and structures of the French bovine STEC O80:H2 strains, it can be said that their virulomes and antimicrobial resistance profiles are similar to those of the previously described human STEC O80:H2 strain RDEx444 [12]. Indeed, all the bovine isolates carried the *stx2d* gene variant associated with more severe infection outcomes [1] and harbored the *eae*-ξ subtype of the intimin-encoding gene characteristic of ST301 strains [32]. Additionally, the studied isolates all harbored a pR444_A-like plasmid combining the conserved regions of the extraintestinal pathogenic *E. coli* pS88 plasmid [34] and a region containing multiple antimicrobial resistance genes. Like other *stx2d*-carrying STEC O80:H2 isolates [12,25], all the French bovine isolates possessed a complete rather than partial set of ExPEC-associated genes, including the salmochelin-encoding gene *iroN* involved in the development of bacteremia [35] but also the *iucC* and *iutA* genes involved in the synthesis of an iron uptake system known to be involved in Avian pathogenic *E. coli* (APEC) pathogenicity [36]. In association with previous virulence factors, the isolates presented herein also carried the enterohemolysin *ehxA* gene frequently associated with enterohemorrhagic *E. coli* (EHEC) clinical strains and located on a pO157-like plasmid described in the above-mentioned reference strain RDEx444. Antimicrobial susceptibility testing revealed resistance to several antimicrobial drug classes of both veterinary and clinical relevance. However, AMR testing did not reveal resistance to azithromycin, which is, to this day, associated with imipenem, the most promising therapeutic option for the treatment of invasive infections associated with STEC O80:H2 [37].

Taken together, these findings support the hypothesis that healthy cattle can act as a reservoir of this emerging STEC-ExPEC heteropathotype and that STEC-ExPEC O80:H2 bovine isolates possess the required virulence-associated genetic background for inducing both HUS and extra-intestinal invasive infections.

Cattle are widely considered a main reservoir of non-pathogenic *E. coli* and STEC strains from other serotypes (both O157:H7 and non-O157:H7 STEC), which they can carry in their gastrointestinal tracts and shed in farm environments [17,38]. Known for its genomic plasticity, *E. coli* can acquire virulence and antimicrobial resistance factors through transmissible genetic elements such as plasmids, pathogenicity islands, or bacteriophages [39]. Such mechanisms have already been described multiple times as the starting point of the emergence of new hybrid pathotypes like the German O104:H4 STEC clone involved in a transnational outbreak, a pathotype that combined the virulence properties of both EHEC and enteroaggregative *E. coli* (AEEC) [40]. Recently, in an experimental setting, a Belgian study demonstrated that the highly virulent *Stx2d* phage from STEC O80:H2 could be successfully transferred to other non-pathogenic *E. coli* strains and was stable in these new hosts. The newly transduced strains were able to induce higher mortality rates than the corresponding non-transduced strains in a *Galleria mellonella* larvae model [41]. Also, in Italy, a nation-wide phylogenomic study of human clinical O26:H11 STEC stains identified five isolates harboring pR444_A-associated AMR and virulence genetic features including *hlyF*, *iroN*, *sitA*, and *iucC* [42]. Moreover, such observations concur with previous experimental findings made by the European Reference Laboratory regarding *E. coli* (Istituto Superiore di Sanità, Rome, Italy). Indeed, STEC O26:H11 strain ED1284 was not only able to acquire and maintain the pR444_A plasmid through conjugation but also transfer this plasmid to other *E. coli* isolates [31]. Hence, in the cattle GIT and the farm environment, STEC-ExPEC O80:H2 could act as a reservoir of virulence-associated genes and AMR genetic determinants. Coexistence of this pathotype with other pathogenic or non-pathogenic *E. coli* within such ecological niches could lead to the emergence of newly virulent *E. coli* clones or hypervirulent hybrid clones.

As the investigated bovine strains were recovered from a single cattle farm, only limited conclusions can be drawn, and additional investigations are needed regarding the reservoirs and potential transmission routes of STEC-ExPEC O80:H2. Indeed, in order to confirm the role of healthy cattle as a potential reservoir for STEC-ExPEC O80:H2, further investigations should focus on the sustainability of cattle as a reservoir for this pathotype through conducting long-term follow-ups regarding the shedding of the latter by healthy cattle. Also, surveys including more cattle from diverse geographic and production system backgrounds should be conducted to better assess the weight of the cattle reservoir. In addition, the development of research aimed at assessing the impact of antibiotic administration in cattle on the selection or emergence of new STEC strains is becoming a priority. However, it is important to remember that even if STEC strains are present in cattle and circulate in the farm environment, if correctly implemented, appropriate STEC control measures can limit the contamination of food products.

Nevertheless, such findings, as limited as they are, may have implications in terms of public health and suggest an increased need for better surveillance of this pathotype in food products of animal origin. So far, STEC O80:H2 have rarely been isolated from foodstuffs and two mains hypotheses stand as to why such strains have not been detected at the food processing stage yet: (i) compared to other STEC serogroup, O80:H2 might be less resistant to the environmental conditions encountered during certain food-processing steps and might be stressed and non-culturable or/and (ii) the current detections methods for STEC O80:H2 strains are not adapted and sensitive enough to detect such strains in foodstuffs. It would, therefore, be relevant to properly evaluate the behavior of STEC O80:H2 strains in different types of food products and environments, and it also appears crucial to develop effective detection methods for this pathotype in foodstuffs. Specifically, further research efforts should be directed towards the optimization of the isolation of these strains after the food matrix enrichment step [43].

## 4. Materials and Methods

### 4.1. Identification and Isolation of O80:H2 STEC Strains from Cattle Feces

During an ongoing longitudinal survey primarily investigating the “top 5” STEC stains carried in cattle, fresh cattle feces samples were collected from all 44 animals present on a single cattle farm in France in May 2023. For each sample, 25 g of feces was enriched in 225 mL of modified Tryptone Soya Broth (mTSB, Oxoid, Dardilly, France) supplemented with 16 mg·L^−1^ of novobiocin and incubated for 18–24 h at 41.5 °C. Post incubation, DNA was extracted from a 1 mL portion using a DNA EZ1 tissue kit via an automated extractor (EZ1-BioRobots^®^, Qiagen, Courtaboeuf, France).

The virulence-associated genes *stx2* and *eae* were screened using a real-time-PCR-based approach adapted from the ISO/TS 13136:2012 [43]. Samples that were positive for the simultaneous presence of *stx2* and *eae* were plated onto CHROMagar™ STEC agar (CHROMagar, Paris, France) and cefixime-tellurite-sorbitol-MacConkey agar (CT-SMAC; Oxoid, Dardilly France) and incubated for 18–24 h at 37 °C. Up to 20 colonies were subjected to rapid DNA extraction via boiling for 15 min and tested for the presence of *stx2* and *eae* using real-time PCR as described above. Isolates positive for both genes underwent further investigations. In addition, a multiplex PCR combining the searching of both the wzy_O80_ gene characteristic of the O80 serogroup and the 70 mel sequence was carried out. This 70 mel sequence is an insertion of a 70 pb sequence (70 mel) associated with the loss of metabolic function due to the deletion of the entire melibiose operon. This genetic scar is shared by all O80:H2 STEC strains belonging to the sequence type ST301 and responsible for most HUS cases in Europe [44]. When the two targets were detected, the corresponding fliC_H2_ allele was characterized using PCR, as previously described [45].

### 4.2. Bacterial Strains

STEC O80:H2 isolates A10AV1, A13P112V1, A15P129V1, A23P11V1, and A29P14V1 were isolated from 5 healthy cows, and isolates VX1P21V1 and VX4P17V1 were obtained from 2 healthy calves from a single cattle farm in May 2023.

Also, 5 human clinical strains isolated between 2022 and 2023 were provided by the University Hospital Robert-Debré (part of the French National Reference Center for *E. coli*) for sequencing and genomic comparison with the above-mentioned bovine isolates.

### 4.3. DNA Extraction and Whole-Genome Sequencing

Bacterial genomic DNA of all 7 bovine isolates was extracted from Brain–Heart Infusion overnight cultures using the Promega^®^ Wizard Genomic DNA purification kit, which was used according to the manufacturer’s instructions (Promega, Charbonnière-les-bains, France). Quality and quantity control procedures for the extracted DNA included the determination of concentrations using the Qubit™ dsDNA HS 1X kit (Thermo Fisher Scientific, Illkirch, France) and purity screening using a NanoDrop One™ microvolume spectrophotometer (Thermo Fisher Scientific, Waltham, MA, USA). Whole-genome sequencing was outsourced to the Novogene sequencing platform (Novogene, Cambridge, UK) and performed using their in-house protocol via an Illumina NovaSeq 6000 (Illumina, San Diego, CA, USA) with a paired-end 150 bp and 100× average coverage sequencing strategy.

Also, to obtain a high-quality contiguous assembled genome enabling high-quality in-depth characterization, 1 isolate (A13P112V1 which is representative of the 7 isolated strains based on phylogenetic analysis) was submitted to complementary long-read Oxford Nanopore whole-genome sequencing. Briefly, high-molecular-weight DNA extraction was performed using the Wizard^®^ HMW DNA Extraction kit from Promega (Promega, Charbonnière-les-bains, France). Libraries were prepared using the Rapid Barcoding kit (SQK-RBK004) and sequenced using a MinION Mk1B device (Oxford Nanopore Technologies, Oxford, UK).

Total DNA from the 5 French human isolates provided by the French NRC (University Hospital Robert-Debré) was extracted, and quality was assessed according to the above-mentioned process. Sequencing libraries were prepared from the extracted DNA using the Nextera XT DNA Library prep kit, and whole-genome sequencing was performed in-house at the FNRL using the iSeq 100 Illumina platform.

### 4.4. Genomic Characterization of 7 French Bovine and 5 Human STEC Strains

Sequencing reads from the 7 isolates were analyzed using the Shiga-toxin-producing *E. coli* dedicated workflow on the Sciensano Galaxy Platform [46] (https://galaxy.sciensano.be/; STEC pipeline 1.0; accessed on 28 September 2023). Sequentially, quality checks were performed using FastQC v.0.12.1 [47], and sequencing reads were quality-trimmed using Trimmomatic v.0.38 [48]. Genomes were then assembled using SPAdes v 3.13.0 [22], and the assembled sequences were subjected to in silico genotyping. Raw reads and assembled sequences obtained in this study were deposited in the NCBI database under BioProject PRJNA1086868.

Briefly, strains were serotyped using SerotypeFinder v.2.0.1 [49]. Virulence genes were detected using the *E. coli* database from VirulenceFinder v.2.0.5 [50]; plasmid replicons were detected using PlasmidFinder v.2.1.1 [51]. Antimicrobial-resistance-associated genes and chromosomal mutations mediating AMR were identified using the Resfinder tool available on the Center for Genomic Epidemiology (CGE) online platform (http://genepi.food.dtu.dk/resfinder; accessed on 11 October 2023) [52,53]. Multi-locus sequence typing was performed by conducting a BLASTN search for seven housekeeping genes (*adk*, *fumC*, *gyrB*, *icd*, *mdh*, *purA*, and *recA*), and sequence types were assigned according to the Warwick Medical School scheme [54].

For in-depth strain characterization, long reads and short reads from A13P112V1 were submitted to hybrid assembly using the Unicycler tool available on the galaxy Europe platform (https://usegalaxy.eu/; accessed on 21 December 2023) [55], and assembly statistics are shown in Supplementary Table S1. Genome sequences were annotated with the Prokka tool (v.1.14.6) [21] and a multi-fasta file of annotated genes on the RDEx444 reference strain [12] (Genbank accession numbers NZ_QBDM01000001 to NZ_QBDM01000004). Finally, a whole-genome comparison of all the strains with pR444_A via assembled contig alignment using BLAST was conducted on the online Proksee platform [20] (https://proksee.ca/; accessed on 28 December 2023).

### 4.5. Antimicrobial Susceptibility Testing

All isolated bovine isolates were evaluated for their susceptibility to antimicrobial drugs of veterinary and clinical interest, including amoxicillin (AMX), amoxicillin/clavulanic acid (AMC), ceftiofur (TIO), cefoxitin (FOX), cefalexin (CN), cefquinome (CEQ), streptomycin (S), gentamicin (GEN), kanamycin (K), tetracycline (TET), colistin (CL), sulfamethoxazole/trimethoprim (SXT), ciprofloxacin (CIP), nalidixic acid (NAL), flumequine (UB), enrofloxacin (ENO), marbofloxacin (MAR), and azithromycin (AZM), using the disk-diffusion method according to the NF-U47-107 technical specification for Enterobacteriaceae [56]. Resistance or susceptibility profiles were determined according to the Société Française de Microbiologie (SFM) and the European Committee on Antimicrobial Susceptibility testing (EUCAST) guidelines [24].

### 4.6. Core-Genome-Based Phylogenetic Analysis

To investigate the phylogenetic positions of our bovine isolates among O80:H2 STEC strains, additional genome sequences of O80:H2 STEC strains from previous studies and from the NCBI and Enterobase databases (last access 13 December 2023) were included in this study. Among all the sequenced human STEC O80:H2 strains from France available on Enterobase, 19 sequences of strains with distinct HC5 clusters [57] isolated between 2018 and 2019 were downloaded along with 7 genomes from clinical isolates available from the UK available on GenBank.

In addition, assembled sequences from 3 French strains, namely 511-4, H39-78, and H15-66, isolated from dairy products were selected from among strains characterized in a previous French study. Considering their geographic proximity, all bovine and human STEC O80:H2 strains’ sequenced genomes from 2 Swiss studies [25,26] and 1 Belgian study [15] were retrieved for genomic comparison with our strains. A summary of all the sequenced strains used in this study is presented in Table 2. Details regarding all included insolates and their accession numbers are listed in Supplementary Table S1.

The clonal relationships of the 7 isolated bovine O80:H2 strains were determined via core-genome multilocus sequence typing (cgMLST) using the chewBACCA tool v.2.0 [58] available on the external ARIES galaxy platform and using the scheme developed by the INNUENDO project [59], with default settings. A distance matrix showcasing the number of cgMLST allelic differences is available in Supplementary Table S2. Their phylogenetic positions among the 100 additional selected strains were investigated using the same process. The phylogenetic tree was visualized using iTOL [60] (https://itol.embl.de/#; accessed on 22 January 2024).

## Figures and Tables

**Table 1:** Antimicrobial susceptibility profile of STEC O80:H2 bovine isolates from this study.

Tested Antibiotics	Critical Diameters (mm; V ^1^/H ^2^)	Diameter (mm)/Susceptibility Phenotype ^4^						
		A10AV1	A13P112V1	A15P129V1	A23P11V1	A29P14V1	VX1P21V1	VX4P17V1
Amoxicillin	14–21/19	0/R	0/R	0/R	0/R	0/R	0/R	0/R
Amoxicillin/Clavulanic acid	14–21/19–20	20/I	19/I	20/I	17/I	18/I	18/I	17/I
Ceftiofur	18–21/NA ^3^	33/S	26/S	22/S	31/S	30/S	30/S	30/S
Cefoxitin	15–22/18	29/S	27/S	28/S	29/S	30/S	26/S	27/S
Cefalexin	12–18/14	21/S	19/S	20/S	20/S	21/S	21/S	20/S
Cefquinome	19–22/NA	39/S	32/S	34/S	38/S	32/S	36/S	37/S
Streptomycin	13–15/NA	0/R	0/R	0/R	0/R	0/R	0/R	0/R
Gentamicin	15–17/17	21/S	21/S	25/S	27/S	26/S	25/S	26/S
Kanamycin	16–18/NA	0/R	0/R	0/R	0/R	0/R	0/R	0/R
Tetracycline	17–19/17	27/S	0/R	0/R	0/R	0/R	0/R	0/R
Colistin	15–18/NA	21/S	17/I	21/S	22/S	21/S	20/S	20/S
Sulfamethoxazole/Trimethoprim	10–16/11–16	0/R	0/R	0/R	0/R	0/R	0/R	0/R
Ciprofloxacin	NA/14	34/S	25/S	34/S	28/S	35/S	35/S	34/S
Nalidixic acid	15–20/14	0/R	0/R	0/R	0/R	0/R	0/R	0/R
Flumequine	21–25/NA	18/R	15/R	18/R	13/R	16/R	16/R	18/R
Enrofloxacin	19/NA	20/S	19/S	20/S	19/S	21/S	20/S	20/S
Marbofloxacin	19/NA	29/S	20/S	29/S	19/S	24/S	25/S	25/S
Azithromycin	NA/12	20/S	17/S	20/S	21/S	22/S	20/S	20/S

^1^ According to the veterinary AMR-testing guidelines of the French Microbiology Society (SFM) [23]. ^2^ According to the SFM-EUCAST antimicrobial susceptibility testing guidelines [24]. ^3^ Not Applicable. ^4^ Susceptibility phenotypes: S = susceptible; I = intermediate; R = resistant.

**Table 2:** Summary of all sequenced isolates included in the phylogenetic analysis.

Host/ Source of Isolation	Location	Number of Isolates	Isolation Date Ranges	References
Human	France	25	2016–2023	[12]; Enterobase, this study.
	Switzerland	37	2003–2019	[25,26]
	Belgium	19	2008–2019	[15]
	UK	7	2013–2020	Enterobase; NCBI GenBank
Cattle	France	7	2023	This study.
	Belgium	9	1987–2018	[15]
Foodstuffs	France	3	2013–2014	[12]

## Data Availability

All data are available in this publication and in the Supplementary Materials.

## Supplementary Materials

The supporting information can be downloaded at: https://www.mdpi.com/article/10.3390/ijms25105428/s1.

## References

[1] Fuller C.A., Pellino C.A., Flagler M.J., Strasser J.E., Weiss A.A. (2011). Shiga Toxin Subtypes Display Dramatic Differences in Potency. Infect. Immun..

[2] ANSES (2023). Avis de l’Agence Nationale de Sécurité Sanitaire de L’alimentation, de L’environnement et du Travail Relatif à la Définition des Souches Pathogènes d’Escherichia coli Productrices de Shigatoxines.

[3] Kaper J.B., Nataro J.P., Mobley H.L.T. (2004). Pathogenic *Escherichia coli*. Nat. Rev. Microbiol..

[4] Lai Y., Rosenshine I., Leong J.M., Frankel G. (2013). Intimate host attachment: Enteropathogenic and enterohaemorrhagic *Escherichia coli*: Intimate EPEC and EHEC adhesion. Cell. Microbiol..

[5] EFSA Panel on Biological Hazards (BIOHAZ) (2013). Scientific Opinion on VTEC-seropathotype and scientific criteria regarding pathogenicity assessment. EFSA J..

[6] Mariani-Kurkdjian P., Lemaître C., Bidet P., Perez D., Boggini L., Kwon T., Bonacorsi S. (2014). Haemolytic-uraemic syndrome with bacteraemia caused by a new hybrid *Escherichia coli* pathotype. New Microbes New Infect..

[7] Soysal N., Mariani-Kurkdjian P., Smail Y., Liguori S., Gouali M., Loukiadis E., Fach P., Bruyand M., Blanco J., Bidet P. (2016). Enterohemorrhagic *Escherichia coli* Hybrid Pathotype O80:H2 as a New Therapeutic Challenge. Emerg. Infect. Dis..

[8] Bruyand M., Mariani-Kurkdjian P., Le Hello S., King L.-A., Van Cauteren D., Lefevre S., Gouali M., Jourdan-da Silva N., Mailles A., Donguy M.-P. (2019). Paediatric haemolytic uraemic syndrome related to Shiga toxin-producing *Escherichia coli*, an overview of 10 years of surveillance in France, 2007 to 2016. Eurosurveillance.

[9] Jones G., Mariani-Kurkdjian P., Cointe A., Bonacorsi S., Lefèvre S., Weill F.-X., Le Strat Y. (2023). Sporadic Shiga Toxin–Producing *Escherichia coli*—Associated Pediatric Hemolytic Uremic Syndrome, France, 2012–2021. Emerg. Infect. Dis..

[10] European Food Safety Authority, European Centre for Disease Prevention and Control (2022). The European Union One Health 2021 Zoonoses Report. EFSA J..

[11] Blanco M., Blanco J.E., Mora A., Dahbi G., Alonso M.P., González E.A., Bernárdez M.I., Blanco J. (2004). Serotypes, Virulence Genes, and Intimin Types ofShiga Toxin (Verotoxin)-Producing *Escherichia coli* Isolatesfrom Cattle in Spain and Identification of a New Intimin VariantGene(*eae-*ξ). J. Clin. Microbiol..

[12] Cointe A., Birgy A., Mariani-Kurkdjian P., Liguori S., Courroux C., Blanco J., Delannoy S., Fach P., Loukiadis E., Bidet P. (2018). Emerging Multidrug-Resistant Hybrid Pathotype Shiga Toxin–Producing *Escherichia coli* O80 and Related Strains of Clonal Complex 165, Europe. Emerg. Infect. Dis..

[13] Thiry D., Saulmont M., Takaki S., De Rauw K., Duprez J.-N., Iguchi A., Piérard D., Mainil J.G. (2017). Enteropathogenic *Escherichia coli* O80:H2 in Young Calves with Diarrhea, Belgium. Emerg. Infect. Dis..

[14] De Rauw K., Thiry D., Caljon B., Saulmont M., Mainil J., Piérard D. (2019). Characteristics of Shiga toxin producing- and enteropathogenic *Escherichia coli* of the emerging serotype O80:H2 isolated from humans and diarrhoeic calves in Belgium. Clin. Microbiol. Infect..

[15] Habets A., Crombé F., Nakamura K., Guérin V., De Rauw K., Piérard D., Saulmont M., Hayashi T., Mainil J.G., Thiry D. (2021). Genetic characterization of Shigatoxigenic and enteropathogenic *Escherichia coli* O80:H2 from diarrhoeic and septicaemic calves and relatedness to human Shigatoxigenic *E. coli* O80:H2. J. Appl. Microbiol..

[16] Rodwell E.V., Vishram B., Smith R., Browning L., Smith-Palmer A., Allison L., Holmes A., Godbole G., McCarthy N., Dallman T.J. (2021). Epidemiology and genomic analysis of Shiga toxin-producing *Escherichia coli* clonal complex 165 in the UK. J. Med. Microbiol..

[17] World Health Organization (2022). Control Measures for Shiga Toxin-Producing Escherichia coli (STEC) Associated with Meat and Dairy Products.

[18] Ministère de L’agriculture et de la Souveraineté Alimentaire (MASA) Plans de Surveillance et Plans de Contrôle. https://agriculture.gouv.fr/plans-de-surveillance-et-de-controle.

[19] Yoshida H., Bogaki M., Nakamura M., Nakamura S. (1990). Quinolone resistance-determining region in the DNA gyrase gyrA gene of *Escherichia coli*. Antimicrob. Agents Chemother..

[20] Grant J.R., Enns E., Marinier E., Mandal A., Herman E.K., Chen C., Graham M., Van Domselaar G., Stothard P. (2023). Proksee: In-depth characterization and visualization of bacterial genomes. Nucleic Acids Res..

[21] Seemann T. (2014). Prokka: Rapid prokaryotic genome annotation. Bioinformatics.

[22] Bankevich A., Nurk S., Antipov D., Gurevich A.A., Dvorkin M., Kulikov A.S., Lesin V.M., Nikolenko S.I., Pham S., Prjibelski A.D. (2012). SPAdes: A New Genome Assembly Algorithm and Its Applications to Single-Cell Sequencing. J. Comput. Biol..

[23] SFM Comité de l’antibiogramme de la Société Française de Microbiologie—Recommandations Vétérinaires 2023. https://www.sfm-microbiologie.org/wp-content/uploads/2023/06/CASFM_VET2023.pdf.

[24] SFM, EUCAST Comité de l’antibiogramme de la Société Française de Microbiologie—Recommandations 2023. https://www.sfm-microbiologie.org/boutique/comite-de-lantibiograme-de-la-sfm-casfm/.

[25] (2007). InVS Epidémie d’infections à E.coli producteurs de shiga-toxines non O157 liée à la consomation de camembert au lait cru, Nord Ouest de la France, Octobre-Décembre 2005. https://www.santepubliquefrance.fr/regions/normandie/documents/rapport-synthese/2007/epidemie-d-infections-a-e.-coli-producteurs-de-shiga-toxines-non-0157-liee-a-la-consommation-de-camembert-au-lait-cru-nord-ouest-de-la-france-oct.

[26] Espié E., Grimont F., Mariani-Kurkdjian P., Bouvet P., Haeghebaert S., Filliol I., Loirat C., Decludt B., Minh N.N.T., Vaillant V. (2008). Surveillance of Hemolytic Uremic Syndrome in Children Less Than 15 Years of Age, a System to Monitor O157 and Non-O157 Shiga Toxin-Producing *Escherichia coli* Infections in France, 1996–2006. Pediatr. Infect. Dis. J..

[27] Wijnsma K.L., Schijvens A.M., Rossen J.W.A., Kooistra-Smid A.M.D., Schreuder M.F., van de Kar N.C.A.J. (2017). Unusual severe case of hemolytic uremic syndrome due to Shiga toxin 2d-producing *E. coli* O80:H2. Pediatr. Nephrol..

[28] Nüesch-Inderbinen M., Cernela N., Wüthrich D., Egli A., Stephan R. (2018). Genetic characterization of Shiga toxin producing *Escherichia coli* belonging to the emerging hybrid pathotype O80:H2 isolated from humans 2010–2017 in Switzerland. Int. J. Med. Microbiol..

[29] Ingelbeen B., Bruyand M., Mariani-Kurkjian P., Le Hello S., Danis K., Sommen C., Bonacorsi S., de Valk H. (2018). Emerging Shiga-toxin-producing *Escherichia coli* serogroup O80 associated hemolytic and uremic syndrome in France, 2013-2016: Differences with other serogroups. PLoS ONE.

[30] Gigliucci F., van Hoek A.H.A.M., Chiani P., Knijn A., Minelli F., Scavia G., Franz E., Morabito S., Michelacci V. (2021). Genomic Characterization of *hlyF* -positive Shiga Toxin–Producing *Escherichia coli*, Italy and the Netherlands, 2000–2019. Emerg. Infect. Dis..

[31] Cointe A., Bizot E., Delannoy S., Fach P., Bidet P., Birgy A., Weill F.-X., Lefèvre S., Mariani-Kurkdjian P., Bonacorsi S. (2021). Emergence of New ST301 Shiga Toxin-Producing *Escherichia coli* Clones Harboring Extra-Intestinal Virulence Traits in Europe. Toxins.

[32] Ikeda R., Nakamura K., Saulmont M., Habets A., Duprez J.-N., Korsak N., Hayashi T., Thiry D., Mainil J.G. (2023). *Escherichia coli* O80 in Healthy Cattle: Absence of Shigatoxigenic and Enteropathogenic *E. coli* O80:H2 and (Phylo) Genomics of Non-Clonal Complex 165 *E. coli* O80. Microorganisms.

[33] Peigne C., Bidet P., Mahjoub-Messai F., Plainvert C., Barbe V., Médigue C., Frapy E., Nassif X., Denamur E., Bingen E. (2009). The Plasmid of *Escherichia coli* Strain S88 (O45:K1:H7) That Causes Neonatal Meningitis Is Closely Related to Avian Pathogenic *E. coli* Plasmids and Is Associated with High-Level Bacteremia in a Neonatal Rat Meningitis Model. Infect. Immun..

[34] Nègre V.L., Bonacorsi S., Schubert S., Bidet P., Nassif X., Bingen E. (2004). The Siderophore Receptor IroN, but Not the High-Pathogenicity Island or the Hemin Receptor ChuA, Contributes to the Bacteremic Step of *Escherichia coli* Neonatal Meningitis. Infect. Immun..

[35] Ling J., Pan H., Gao Q., Xiong L., Zhou Y., Zhang D., Gao S., Liu X. (2013). Aerobactin Synthesis Genes iucA and iucC Contribute to the Pathogenicity of Avian Pathogenic *Escherichia coli* O2 Strain E058. PLoS ONE.

[36] Stevens M.J.A., Cernela N., Müller A., Nüesch-Inderbinen M., Stephan R. (2021). Draft Genome Sequences of 19 Clinical *stx*-Harboring *Escherichia coli* O80:H2 Strains. Microbiol. Resour. Announc..

[37] Cointe A., Birgy A., Bridier-Nahmias A., Mariani-Kurkdjian P., Walewski V., Lévy C., Cohen R., Fach P., Delannoy S., Bidet P. (2020). *Escherichia coli* O80 hybrid pathotype strains producing Shiga toxin and ESBL: Molecular characterization and potential therapeutic options. J. Antimicrob. Chemother..

[38] Withenshaw S.M., Smith R.P., Davies R., Smith A.E.O., Gray E., Rodgers J. (2022). A systematized review and qualitative synthesis of potential risk factors associated with the occurrence of non-O157 Shiga toxin-producing *Escherichia coli* (STEC) in the primary production of cattle. Compr. Rev. Food Sci. Food Saf..

[39] Tawfick M.M., Elshamy A.A., Mohamed K.T., El Menofy N.G. (2022). Gut Commensal Escherichia coli, a High-Risk Reservoir of Transferable Plasmid-Mediated Antimicrobial Resistance Traits. Infect. Drug Resist..

[40] Denamur E. (2011). The 2011 Shiga toxin-producing *Escherichia coli* O104:H4 German outbreak: A lesson in genomic plasticity. Clin. Microbiol. Infect..

[41] Habets A., Antoine C., Wagemans J., Vermeersch M., Laforêt F., Diderich J., Lavigne R., Mainil J., Thiry D. (2022). Impact of Shiga-toxin encoding gene transduction from O80:H2 Shiga toxigenic *Escherichia coli* (STEC) on non-STEC strains. Sci. Rep..

[42] Michelacci V., Montalbano Di Filippo M., Gigliucci F., Arancia S., Chiani P., Minelli F., Roosens N.H.C., De Keersmaecker S.C.J., Bogaerts B., Vanneste K. (2022). Population Analysis of O26 Shiga Toxin-Producing *Escherichia coli* Causing Hemolytic Uremic Syndrome in Italy, 1989–2020, Through Whole Genome Sequencing. Front. Cell. Infect. Microbiol..

[43] AFNOR (2012). Méthode Basée sur la PCR en Temps Réel Pour la Détection des Micro-Organismes Pathogènes dans les Aliments: Méthode Horizontale pour la Étection des STEC et la Détermination des Sérogroupes O157, O111, O26, O103, O145.

[44] Bizot E., Cointe A., Smadja N., Sergentet D., Lefèvre S., Weill F.-X., Levy C., Cohen R., Mariani-Kurkdjian P., Bonacorsi S. (2022). Improved Molecular Diagnosis and Culture of the Emerging Heteropathotype Enterohemorrhagic *Escherichia coli* O80:H2 Using Its Non-Melibiose-Fermenting and Antibiotic-Resistance Properties. J. Clin. Microbiol..

[45] Madic J., Peytavin de Garam C., Vingadassalon N., Oswald E., Fach P., Jamet E., Auvray F. (2010). Simplex and multiplex real-time PCR assays for the detection of flagellar (H-antigen) fliC alleles and intimin (eae) variants associated with enterohaemorrhagic *Escherichia coli* (EHEC) serotypes O26:H11, O103:H2, O111:H8, O145:H28 and O157:H7: Real-time PCR for the detection of major EHEC serotypes. J. Appl. Microbiol..

[46] Bogaerts B., Nouws S., Verhaegen B., Denayer S., Van Braekel J., Winand R., Fu Q., Crombé F., Piérard D., Marchal K. (2021). Validation strategy of a bioinformatics whole genome sequencing workflow for Shiga toxin-producing *Escherichia coli* using a reference collection extensively characterized with conventional methods. Microb. Genomics.

[47] Andrews S. FastQC: A Quality Control Tool for High Throughput Sequence Data. http://www.bioinformatics.babraham.ac.uk/projects/fastqc.

[48] Bolger A.M., Lohse M., Usadel B. (2014). Trimmomatic: A flexible trimmer for Illumina sequence data. Bioinformatics.

[49] Joensen K.G., Tetzschner A.M.M., Iguchi A., Aarestrup F.M., Scheutz F. (2015). Rapid and Easy *In Silico* Serotyping of *Escherichia coli* Isolates by Use of Whole-Genome Sequencing Data. J. Clin. Microbiol..

[50] Joensen K.G., Scheutz F., Lund O., Hasman H., Kaas R.S., Nielsen E.M., Aarestrup F.M. (2014). Real-Time Whole-Genome Sequencing for Routine Typing, Surveillance, and Outbreak Detection of Verotoxigenic Escherichia coli. J. Clin. Microbiol..

[51] Carattoli A., Hasman H., de la Cruz F. (2020). PlasmidFinder and In Silico pMLST: Identification and Typing of Plasmid Replicons in Whole-Genome Sequencing (WGS). Horizontal Gene Transfer.

[52] Bortolaia V., Kaas R.S., Ruppe E., Roberts M.C., Schwarz S., Cattoir V., Philippon A., Allesoe R.L., Rebelo A.R., Florensa A.F. (2020). ResFinder 4.0 for predictions of phenotypes from genotypes. J. Antimicrob. Chemother..

[53] Camacho C., Coulouris G., Avagyan V., Ma N., Papadopoulos J., Bealer K., Madden T.L. (2009). BLAST+: Architecture and applications. BMC Bioinformatics.

[54] Wirth T., Falush D., Lan R., Colles F., Mensa P., Wieler L.H., Karch H., Reeves P.R., Maiden M.C.J., Ochman H. (2006). Sex and virulence in *Escherichia coli*: An evolutionary perspective. Mol. Microbiol..

[55] Wick R.R., Judd L.M., Gorrie C.L., Holt K.E. (2017). Unicycler: Resolving bacterial genome assemblies from short and long sequencing reads. PLOS Comput. Biol..

[56] Association Française de Normalisation (AFNOR) (2012). Méthodes d’analyse en Santé Animale—Guide de Réalisation des Antibiogrammes par la Méthode de Diffusion en Milieu Gélosé.

[57] Zhou Z., Charlesworth J., Achtman M. (2021). HierCC: A multi-level clustering scheme for population assignments based on core genome MLST. Bioinformatics.

[58] Silva M., Machado M.P., Silva D.N., Rossi M., Moran-Gilad J., Santos S., Ramirez M., Carriço J.A. (2018). chewBBACA: A complete suite for gene-by-gene schema creation and strain identification. Microb. Genomics.

[59] Rossi M., Silva M.S.D., Ribeiro-Gonçalves B.F., Silva D.N., Machado M.P., Oleastro M., Borges V., Isidro J., Viera L., Halkilahti J. (2018). Innuendo Whole Genome and Core Genome Mlst Schemas and Datasets for *Escherichia coli*. Zenodo.

[60] Letunic I., Bork P. (2021). Interactive Tree of Life (iTOL) v5: An online tool for phylogenetic tree display and annotation. Nucleic Acids Res..

